# Adherence to Dietary Recommendations of 7-Year-Old Children from a Birth Cohort in Friuli Venezia Giulia, Italy

**DOI:** 10.3390/nu14030515

**Published:** 2022-01-25

**Authors:** Elisa Giordani, Michela Marinoni, Federica Fiori, Federica Concina, Luca Ronfani, Patrizia Dalmin, Fabio Barbone, Valeria Edefonti, Maria Parpinel

**Affiliations:** 1 Department of Medicine—DAME, University of Udine, Via Colugna 50, 33100 Udine, Italy; elisa.giordani@uniud.it (E.G.); michela.marinoni@uniud.it (M.M.); fabio.barbone@uniud.it (F.B.); maria.parpinel@uniud.it (M.P.); 2 Clinical Epidemiology and Public Health Research Unit, Institute for Maternal and Child Health—IRCCS “Burlo Garofolo”, Via dell’Istria 65/1, 34137 Trieste, Italy; federica.concina@burlo.trieste.it (F.C.); luca.ronfani@burlo.trieste.it (L.R.); patrizia.dalmin@burlo.trieste.it (P.D.); 3 Institute of Hygiene and Clinical Epidemiology, Azienda Sanitaria Universitaria Friuli Centrale, Via Colugna 50, 33100 Udine, Italy; 4 Branch of Medical Statistics, Biometry, and Epidemiology “G.A. Maccacaro”, Department of Clinical Sciences and Community Health, Università degli Studi di Milano, via Venezian 1, 20133 Milan, Italy

**Keywords:** dietary habits, dietary intake, energy and nutrient intake, nutritional adequacy, primary school children, nutrient adequacy ratio, mean adequacy ratio, dietary reference values, food groups, dietary record

## Abstract

Few Italian and European studies have assessed adherence to dietary recommendations in primary school children using dietary records. No Italian studies have provided an index-based nutritional adequacy assessment. We provided a comprehensive overview of dietary intake in 381 7-year-old children from NAC-II cohort study, Friuli Venezia Giulia (Italy). Energy, macro-, and micronutrient intakes were derived from 3-day dietary records. Standard (median and percentage) and index-based (Nutrient Adequacy Ratio (NAR) and Mean Adequacy Ratio (MAR)) approaches were used to evaluate adequacy to Italian dietary reference values at nutrient- and overall-diet-level. Percentage contribution of macronutrients to energy intake (%En) was unbalanced towards total fats and protein. In 25% of children, total fats intake exceeded the reference intake upper limit. In ~63% of children, protein intake was at least doubled in their child-specific population reference intake. Median intakes of sodium (1.7 g/day), saturated fatty acids (12.2 %En), and soluble carbohydrates (19.4 %En) exceeded the suggested dietary target in most (65–84%) children. Inadequacy was also observed for micronutrients, with median NARs ranging from 0.11 (vitamin D) to 0.90 (zinc). The median MAR was 0.75 (0.69–0.79), with 1 indicating optimal overall dietary intake. In conclusion, the enrolled children showed suboptimal intakes of several macro- and micronutrients, in line with Italian and European studies on primary school children. Based on the current findings, public health interventions may be targeted to specific nutrients or subpopulations.

## 1. Introduction

Over recent decades, environmental conditions, including nutrition, have been increasingly recognized to provide short- and long-term effects on health [1,2]. A balanced diet during pregnancy, infancy, and childhood is likely to provide a healthier development over the lifespan [3] and to prevent from the onset of the most common noncommunicable diseases, including obesity [1], type 2 diabetes [4], cardiovascular disease [5], and cancer [6], with childhood obesity itself being one of the major risk factors in the development of other noncommunicable diseases [7,8,9]. In Europe, the prevalence of overweight and obesity in primary school children is still a considerable public health issue. Italy is one of the most affected countries [10], reaching 20.4% and 9.4% of prevalence, respectively [11]. In addition, in children, type 2 diabetes is becoming more common [12], even if it is still an occasional event [13]. A balanced nutritional intake including some key nutrients in childhood, such as polyunsaturated fatty acids (PUFAs), iron, iodine, and vitamin B12, is also important in children’s neurodevelopment early in life, as well as in its short-term maintenance [14,15,16,17,18].

Childhood is a crucial period of growth and development [19]. Entering primary school, children markedly change their lifestyle. European primary school children spend 65% of school time in sedentary activities [20]. Together with modest physical activity levels outside school time [21,22], this suggests monitoring of their dietary patterns may improve our understanding of nutritional status, and the potential associations between diet and diseases [23]. Information on nutrient intakes at the population level may also provide support to organize targeted nutritional programs [24]. To address this goal, good-quality data on the macro- and micronutrient profile of children’s diets are a key element [25].

Different dietary assessment tools have been traditionally used for investigating nutritional habits in children. Among them, food frequency questionnaires (FFQ) are useful to assess the habitual food consumption over long periods in large samples but may introduce errors at individual level for the assessment of energy, macro-, and micronutrient intake. Thus, dietary records (food diaries) or 24-h recalls are usually preferred when the aim is to compare nutrient intakes with country- and age-specific dietary recommendations or estimate mean energy and nutrient intakes [26].

A few studies so far have described nutrient intakes, food sources, and/or adherence to national and international dietary recommendations in primary school children. In Europe, their dietary habits were assessed using 24-h recalls or dietary records obtained from one [27,28] or multiple waves [29,30,31] of existing cohort studies. In Italy, a cohort was established in 2007 to investigate energy and nutrient intakes from a FFQ in 2–10-year-old children from the Lombardy region, in the north of Italy [32]; two time points were available for the description of dietary habits of primary school children (i.e., 8 and 10 years). Other dietary assessments have been carried out in Italian primary school children using 24-h recalls or dietary records too [24,33,34,35,36,37]. Among all the Italian studies, four [32,34,35,36] compared nutrient intakes with the corresponding Italian Dietary Reference Values (DRVs) [38]. The standard approach followed for each nutrient included: 1. comparison of observed mean/median intakes and DRVs; 2. calculation of the percentage of subjects meeting the DRV requirement. To our knowledge, no Italian studies so far have provided an index-based assessment of the nutrient-specific or overall-diet-specific adequacy in primary school children. Firstly introduced in Madden et al. [39], the Nutrient Adequacy Ratio (NAR) expresses an individual’s intake of a nutrient as a percentage (capped at 100%) of the corresponding recommended allowance for that nutrient, given the respondent’s age and sex. Later applied to the pediatric population by Hatløy et al. [40] and more recently by Eldridge [41], NARs provided the basis for the mean adequacy ratio (MAR) index. The MAR quantifies the overall nutritional adequacy of a population based on an individual’s diet using the current recommended allowance for a group of nutrients of interest [40,41].

The aim of the current paper is to provide a comprehensive overview of dietary intake in 7-year-old Italian children from the Northern Adriatic Cohort (NAC-II), Friuli Venezia Giulia, in the northeast of Italy, by following different approaches:

1. Standard evaluation of adequacy to the DRVs [38];
2. Index-based evaluation of adequacy to DRVs at the following levels:
  - Nutrient-level adequacy, through the NAR index;
  - Overall-diet-level adequacy, through the MAR index;
3. Percentage contribution of different food sources to macro- and micronutrient intakes.

## 2. Materials and Methods

### 2.1. Study Population

Between 2007 and 2009, 900 pregnant women were enrolled in the prospective Italian NAC-II study [42], within the framework of the ‘Public health impact of long-term, low-level, mixed element exposure in susceptible population strata’ (PHIME) European Union project [43]. The project included a Mediterranean cohort involving 4 birth cohorts from Italy (NAC-II), Slovenia, Greece, and Croatia, with the aim of investigating the association between low-level mercury exposure from food consumption in pregnancy and child neurodevelopment at 18 months. Overall diet of mother–infant pairs was originally assessed (during pregnancy, and at 18 months of the child) to provide adjustments for potential confounding factors [43]. Within NAC-II, child’s dietary habits were further assessed at 7 years (2014–2016), following an additional extended protocol [44]. Briefly, at the 7-year follow-up, parents of those children tested for the neurodevelopment outcomes at 18 months (*N* = 632) were contacted for further dietary and neurodevelopment evaluation. The current paper considered dietary intakes for the 381 children whose parents filled in the corresponding dietary record at 7 years of age. A comprehensive description of dietary intake at 18 months has been recently published [45].

The study was conducted according to the Declaration of Helsinki and approved by the Ethics Committee of the Institute for Maternal and Child Health IRCCS Burlo Garofolo (CE/V-109-12/04/2010). All participating families were informed and consented to participate to the study.

### 2.2. Parental and Children’s Characteristics

Parents filled in a questionnaire assessing lifestyle of the enrolled 7-year-old children. Socio-demographic characteristics of both parents, including education level, marital status, and citizenship, were obtained from a questionnaire administered at delivery [43].

Children’s height and weight were measured from healthcare staff during the neuropsychological assessment at 7 years [44]. Body mass index (BMI; kg/m^2^) was calculated as: (weight (kg)/height^2^ (m^2^)). Children were categorized as normal weight, underweight, overweight, or obese according to the World Obesity Federation [46] based on International Obesity Task Force (IOTF) BMI cut-offs for thinness, overweight, and obesity [47].

### 2.3. Dietary Assessment

Dietary data were collected using a 3-day dietary record (3-dDR) filled in at home by one parent instructed on how to record type and portion size of the foods consumed by the child. Common kitchen utensils were suggested as an alternative to traditional kitchen scales to measure solids and fluids (e.g., teaspoon, glass); in this case, estimated equivalents in grams were also indicated to the parents. Food fortification or supplement use was not captured within the 3-dDR as the instructions did not suggest collection of this information. Three children filled in the diary for less than 3 days and 4 children filled in the diary for more than 3 days. A researcher’s telephone contact was provided whenever parents need clarification while filling in the 3-dDR. 

Intakes of 39 selected macro- and micronutrients were derived after uploading individual food information from the 3-dDRs in the Microdiet V4.4.1 software (Microdiet software–Downlee Systems Ltd., High, Peak, UK), which contains the Italian “Food Composition Database for Epidemiological Studies in Italy” [48], integrated with information from nutritional labels when needed. Full details of the methodology were published elsewhere [49].

For each nutrient, the Microdiet software provided total intake over the observation period; we calculated mean daily intakes by dividing total intake by the number of collection days. Total energy intake was estimated by summing the mean daily intake of the single macronutrients each multiplied by the corresponding energy conversion factor.

Single food items from the 3-dDRs were classified into 18 food groups obtained after modification of food grouping schemes provided in previous publications of our group [45,50]. To disentangle the main food sources, percentage contribution of the 18 food groups on energy and nutrients was calculated for each child.

All procedures were conducted by a trained food technologist and three nutritionists, who were fully familiar with the management of food composition data, nutritional assessment, food preparation methods, and nutritional labels.

### 2.4. Nutritional Adequacy

Individual nutrient intakes were compared with the DRVs proposed by the Italian Society for Human Nutrition [38], when available. The DRVs include adequate intake (AI), reference intake (RI) range for macronutrients, average requirement (AR), population reference intake (PRI), and suggested dietary target (SDT) for the corresponding nutrient.

For protein intake, the Italian Society for Human Nutrition provided 3 DRVs including RI range, AR, and PRI. Given the preeminent role of AR and PRI for protein and availability of anthropometric information for most of the children (*N* = 350; ~92%), we calculated the AR and PRI child-specific cut-offs using the individual weights and the age-specific DRVs for 7-year-old children, as: AR(protein) (g/day)= 0.8 g/kg weight per day ×∙kg of weight; PRI(protein) (g/day)= 0.98 g/kg weight per day × kg of weight. This integrates information on RI range, which was calculated by difference, as: RI(protein) = 100% − RI(total fats) − RI(available carbohydrates) [38].

We evaluated the adequacy of individual diets at the nutrient- as well as at the overall-diet-level using the NAR and the MAR, respectively [40]. In detail, the NAR is defined as the ratio of each child’s intake to the national DRV for the appropriate age category. The MAR is the sum of all (nutrient-specific) NARs divided by the total number of NARs. As any ratio, a NAR equal to 1 indicates that the corresponding subject meets the requirement fixed for that nutrient. A MAR equal to 1 indicates that the subjects meet the requirements for all the selected nutrients. 

To take into account inadequacy due to excess intake, we extended the approach proposed by Atløy in children [40] to those macro- and micronutrients for which a maximum desirable intake is available. In detail:

- For all micronutrients with one DRV indicating the minimum desirable intake (i.e., AI or AR), we truncated all NARs greater than 1 to 1 so that these nutrients could not compensate those with a NAR lower than 1 in the MAR calculation;
- For the remaining macro- and micronutrients indicating a maximum desirable intake (i.e., RI: protein, available carbohydrates, total fats, monounsaturated fatty acids (MUFAs), total PUFAs, PUFAs ω-3 and ω-6; SDT: soluble carbohydrates, saturated fatty acids (SFAs), sodium, and chloride), we followed suggestions by Hilbig [51] and redefined NARs greater than 1 (inadequate intake by excess) to be equal to: 1 minus the exceeding amount. For example, when the original NAR was equal to 1.15, our modified NAR value is equal to 0.85.

To assess the importance of the individual nutrients in the MAR calculation, we also carried out an influence analysis where the single components were removed one at a time from the MAR definition.

We additionally evaluated nutrient-specific adequacy of protein intake using child-specific AR and PRI.

### 2.5. Statistical Analysis

General characteristics of parents and children were presented as frequency and percentage distribution for categorical variables, and as median, 25th, and 75th centile for continuous variables with a non-normal distribution. Normality assumption was tested for each continuous variable using the Shapiro–Wilk test.

Standard evaluation of nutritional adequacy was carried out using median, 25th and 75th centile and percentage of children meeting the DRV requirements. Sex-specific median, 25th, and 75th centile were also provided and the presence of potential sex differences was investigated using the two-sample Wilcoxon rank-sum (Mann–Whitney) test. Furthermore, we investigated the presence of potential inadequacy in individual protein intakes by comparing the observed intakes (g/day) with the corresponding AR and PRI (g/day) with the two-sample Wilcoxon rank-sum (Mann–Whitney) test. Index-based evaluation of adequacy was based on median, 25th, and 75th centile of NAR and MAR. 

Statistical significance for all tests was set at 0.05. Stata (StataCorp. 2013. Stata Statistical Software: Release 13. StataCorp LP, College Station, TX, USA) was used for all statistical analysis.

## 3. Results

### 3.1. Lifestyle and Anthropometric Characteristics of the Study Population

In total, 381 children (females: 48.3%; males: 51.7%) whose parents filled in 3-dDR were included in the present study. Mother and father’s socio-demographic characteristics at enrollment are reported in Supplementary Table S1. Only 6.3% of the mothers were foreign citizens. Almost 82% of the mothers and ~70% of the fathers had a high school diploma (45.1% and 47.0%, respectively) or a higher educational level (38.6% and 22.0%, respectively).

Children’s lifestyle and anthropometric characteristics at 7 years of age are presented in Table 1.

Children’s median age was 7.1 (7.1–7.2) years. Approximately 72.9% of the children were normal weight, whereas 19.1% and 5.4% were overweight and obese, respectively. Most of the children (79.5%) practiced extra-curricular sport activities from 1 to 3 days per week, with a 15.2% practiced sport more than 4 days per week. Approximately half of the children (45.1%) played videogames less than 1 h per day or never (27.8%), whereas 66.9% watched TV more than 1 h per day (Table 1). During the weekend, the percentage of children playing videogames and watching TV increased in all categories (data not shown).

Mothers spent more time with children during weekdays, but a prevailing caregiver role was also identified for school (43.4%), fathers (38.7%), and grandparents (35.8%); during the weekend fathers spent more time together with the child (data not shown).

### 3.2. Description of Daily Dietary Nutrient Intake and Standard Comparison with the Italian Dietary Reference Values

#### 3.2.1. Energy and Macronutrients

Overall and sex-specific descriptive statistics (median, 25th–75th centile) of the observed intakes of energy and macronutrients per day are reported in Table 2, together with the corresponding Italian DRVs. The Italian DRVs include RI range, SDT, or AI, as applicable [38].

The median daily energy intake of the overall sample was 1503.0 kcal (1336.2–1668.0), with a statistically significant difference by sex (*p* < 0.05): females tended to have a lower intake with a median of 1466.6 kcal (1256.8–1639.3) compared to 1530.0 kcal (1379.3–1699.3) for males.

Overall, the percentage contribution of protein, total fats, and available carbohydrates to daily energy intake (%En) was found to be in line with the recommendations. The median %En from total fats (31.3 %En; 27.4–35.1) was close to the upper limit of the RI range (20–35 %En). Conversely, the median %En from available carbohydrates (51.8 %En; 48.3–56.6) was closer to the lower limit of the recommendations (45–60%). A statistically significant difference was observed for available carbohydrates (g/day) between females and males (*p* < 0.05) (females: 189.3; 153.0–215.8 vs. males: 204.1; 172.0–230.4). Based on the %En of total fats and available carbohydrates, the median %En of protein (14.8 %En; 13.2–16.5) was at the middle point of the RI range (12–18 %En). Furthermore, the median %En from total PUFAs, PUFAs ω-6, and PUFAs ω-3 were below the lower limit of the RI range. Within the PUFAs dietary profile, median intake of the sum of eicosapentaenoic acid (EPA) and docosahexaenoic acid (DHA) (mg/day) was below the AI. Higher %En from total PUFAs and PUFAs ω-6 were observed in females than in males (*p* < 0.05).

In median, soluble carbohydrates and SFAs intakes exceeded the SDT (<15 %En and <10 %En, respectively) in the overall sample: 19.4 %En (16.4–23.0) for soluble carbohydrates and 12.2 %En (10.6–14.0) for SFAs.

The median fiber intake (7.0 g/1000 kcal per day; 5.7–8.7) did not reach the AI (8.4 g/1000 kcal per day).

#### 3.2.2. Protein Intake: Comparison with Average Requirement and Population Reference Intake

Providing a focus on protein intake, 100% of the enrolled children reached their AR;

99.7% of the children reached their PRI too, with only one child having an intake between AR and PRI. However, 63% of the children showed an observed protein intake 2–4 times higher than their PRI.

**Figure 1:**
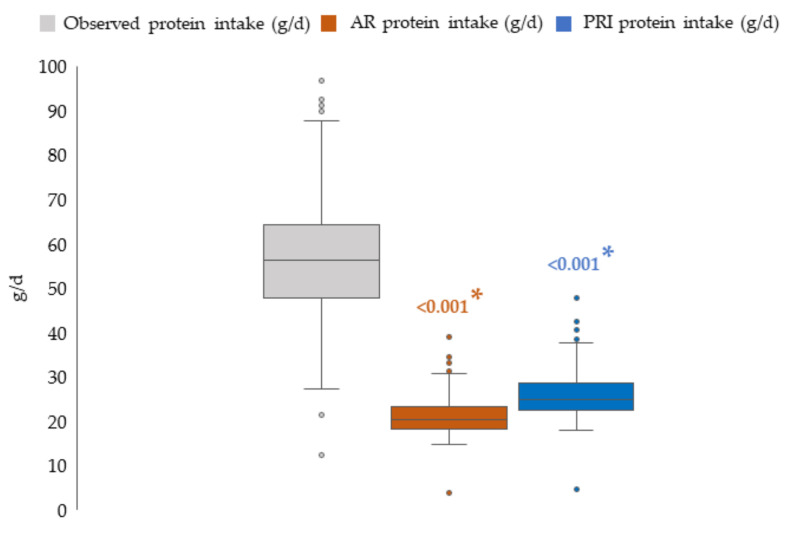
Box-and-whiskers plots comparing the observed protein intake of children and their protein dietary reference values. NAC-II, 2014-2016 (*N* = 381). Each child’s (*N* = 350) protein requirement was estimated using Average Requirement (AR = 0.8 g/kg of weight per day) and Population Reference Intake (PRI = 0.98 g/kg of weight per day) for 7-year-old children. The bottom and top edge of each box represent the 25th and 75th centile (interquartile range); the line within each box represents the median; the ends of the bottom and top whiskers represent the minimum and maximum values and the circles represent outliers. Abbreviation: d, day. The two-sample Wilcoxon rank-sum (Mann–Whitney) test was applied to detect any statistical differences between observed and estimated intakes (AR and PRI). * *p* < 0.05.

Figure 1 shows a comparison between individual-level distributions of observed (i.e., from the 3-dDR) and required (i.e., expressed as their age-specific AR and PRI) intakes for our 7-year-old children. In the absence of sex-specific differences in observed intakes, this analysis was carried out on the overall sample of children. The distribution of the observed protein intake modestly overlapped with that of the corresponding required intakes (*p*-values from the two-sample Wilcoxon rank-sum (Mann–Whitney) test for observed vs. AR intake and observed vs. PRI intake <0.0001). Corresponding summary statistics expressed as median (25th–75th centile) were equal to: observed intake: 56.2 g/day (47.7–64.3) vs. AR intake: 20.4 g/day (18.3–23.3), and PRI intake: 25.0 g/day (22.4–28.6). 

#### 3.2.3. Micronutrients

Overall and sex-specific descriptive statistics (median, 25th–75th centile) of the observed intakes of micronutrients per day are reported in Table 3, together with the corresponding Italian DRVs. The Italian DRVs include AI, SDT, AR, or PRI, as applicable [38].

Median intakes of potassium, magnesium, manganese, iodine, pantothenic acid, biotin, and vitamin E were below the corresponding AI. Median sodium intake (1.7 g/day; 1.3–2.1) reached the AI (1.1 g/day), but it exceeded the SDT (1.5 g/day).

Moreover, median intakes of calcium, chloride, magnesium, zinc, selenium, folate, and vitamin D were below the AR and consequently the PRI. Median intakes of phosphorus, iron, copper, vitamin B1, and niacin reached the corresponding AR, but not the PRI Finally, median intakes of vitamin B2, vitamin B6, vitamin B12, vitamin A, and vitamin C reached both their AR and PRI.

Significantly higher intakes were observed in males as compared to females for calcium, phosphorus, zinc, and vitamin B2 (*p* < 0.05, Table 3).

### 3.3. Index-Based Evaluation of Diet Adequacy to Dietary Reference Values

#### 3.3.1. Nutrient-Level Adequacy

**Figure 2:**
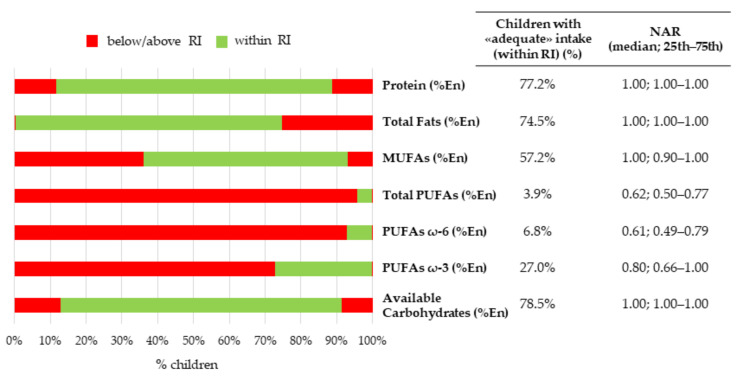
Nutritional adequacy of macronutrients relative to the reference intake. NAC-II, 2014–2016 (*N* = 381). NAR was based on the RI range. Children having intakes equal to the cut-off values were considered to be adequate for that specific nutrient.

**Figure 3:**
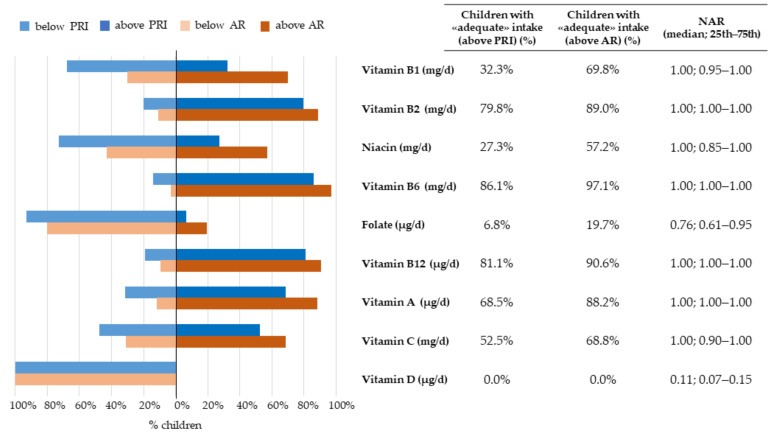
Nutritional adequacy of micronutrients (i.e., vitamins) relative to the average requirement and population reference intake. NAC-II, 2014–2016 (*N* = 381). NAR was based on the AR. Children having intakes equal to the cut-off value were considered to be adequate for that specific nutrient.

**Figure 4:**
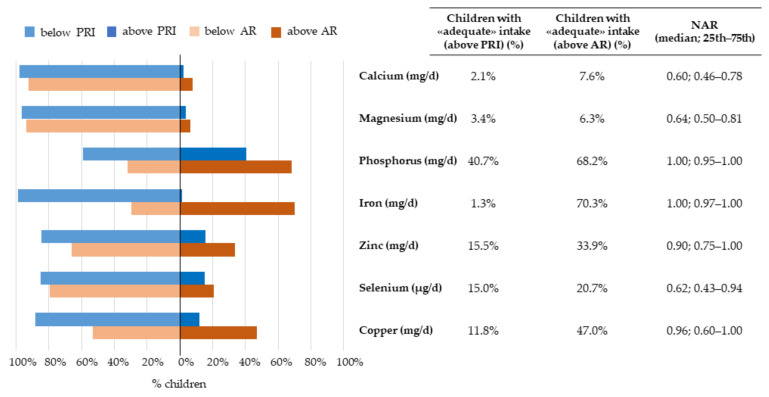
Nutritional adequacy of micronutrients (i.e., minerals) relative to the average requirement and population reference intake. NAC-II, 2014–2016 (*N* = 381). NAR was based on the AR. Children having intakes equal to the cut-off value were considered to be adequate for that specific nutrient.

**Figure 5:**
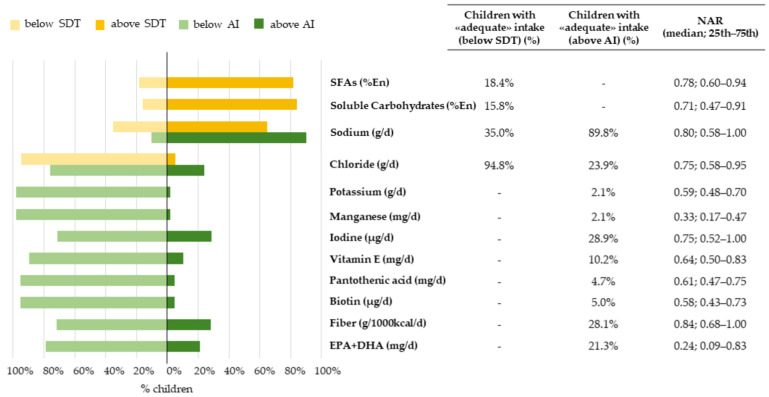
Nutritional adequacy of macro- and micronutrients, relative to the adequate intake and suggested dietary target. NAC-II, 2014–2016 (*N* = 381). NAR was based on the SDT and AI Children having intakes equal to the AI cut-off value were considered to be adequate for that specific nutrient, while children having intake equal to the SDT cut-off value were considered to be inadequate.

The proportion of children with a nutrient intake above, below, or within the recommendations, together with the corresponding median NARs, were presented in Figure 2, Figure 3, Figure 4 and Figure 5.

The RI lower limits for total fats (%En), protein (%En), and available carbohydrates (%En) were reached by 74.5%, 77.2%, and 78.5% of children, respectively. This was confirmed by median NARs being all equal to 1.00 (1.00–1.00)—i.e., the ideal cut-off for nutrient adequacy—for all the previous nutrients (Figure 2).

Approximately 96%, 93%, and 73% of children did not reach the RI lower limit for total PUFAs (%En), PUFA ω-6 (%En), and PUFA ω-3 (%En), respectively. The corresponding median NARs were far from 1 and equal to 0.62 (0.50–0.77), 0.61 (0.49–0.79), and 0.80 (0.66–1.00), respectively, thus suggesting a substantial inadequacy (Figure 2).

In addition, ~80% of children had intakes of vitamin B2, vitamin B6, vitamin B12, and vitamin A above the AR; the corresponding median NAR was equal to 1.00, with the 25th centile already reaching 1. Almost 70% of children had intakes of vitamin B1 and vitamin C above the AR, with median NARs of 1.00, but the 25th centile reached 0.95 and 0.90, respectively. Less than 20% of children had an intake above the AR for folate, with a median NAR of 0.76 (0.61–0.95). No child had an intake above the AR for vitamin D, with a median NAR as low as 0.11 (0.07–0.15) (Figure 3).

Furthermore, almost 70% of children had an intake of iron and phosphorus above the AR, with median NARs of 1.00 (0.97–1.00) and 1.00 (0.95–1.00), respectively. However, only 1% of children had an iron intake above the PRI cut-off. Regarding selenium, zinc, and copper, 20.7%, 33.9%, and 47.0% of children had an intake above the corresponding nutrient-specific AR, respectively; the median NARs were 0.62 (0.43–0.94), 0.90 (0.75–1.00), and 0.96 (0.60–1.00), respectively, thus indicating that the observed intakes for zinc and copper were closer to the AR than selenium. The AR for calcium and magnesium was reached by less than 10% of the children (NARs equal to 0.60 and 0.64, respectively) (Figure 4).

Finally, for fiber, iodine, chloride, and EPA + DHA intake the AI was similarly reached by 20–30% of the children, but the median NARs varied from 0.24 (0.09–0.83, EPA + DHA) to 0.84 (0.68–1.00, fiber). Less than 10% of children had an intake of potassium, manganese, pantothenic acid, and biotin above the AI; the corresponding median NARs varied from 0.33 (0.50–0.81, manganese) to 0.64 (0.50–0.83, vitamin E) (Figure 5).

Overall, 90% of the children had a sodium intake above the AI; however, only 35% had an intake not exceeding the SDT cut-off value, as reflected by a median NAR smaller than 1 (NAR: 0.80, 0.58–1.00). Even if most children exceeded the SDT for SFAs and soluble carbohydrates (81.6% and 84.2%, respectively), the corresponding median NARs were 0.78 (0.60–0.94) and 0.71 (0.47–0.91), thus witnessing a modest distance of observed intakes from the SDT (Figure 5). However, when analyzing individual intakes with more stringent cut-offs, no child from our sample had a soluble carbohydrates intake <5 %En, 4 children only were <10 %En, vs. 60 (15.8%) who were <15 %En, which corresponds to the Italian SDT. Finally, 57 children (15.0%) had a soluble carbohydrates intake exceeding the recommended 25% of energy intake [38] (data not shown).

#### 3.3.2. Overall-Diet-Level Adequacy

In our population, no children reached the optimal MAR value of 1.00, targeting adequacy on all the available nutrients. Overall, the median MAR was 0.75 (0.69–0.79). In the influence analysis, median values of the MAR ranged from 0.74 to 0.76, after removal of one component at a time. No statistically significant differences were found in median MAR values between females and males.

### 3.4. Sources of Nutrient Intakes: Food Groups

**Figure 6:**
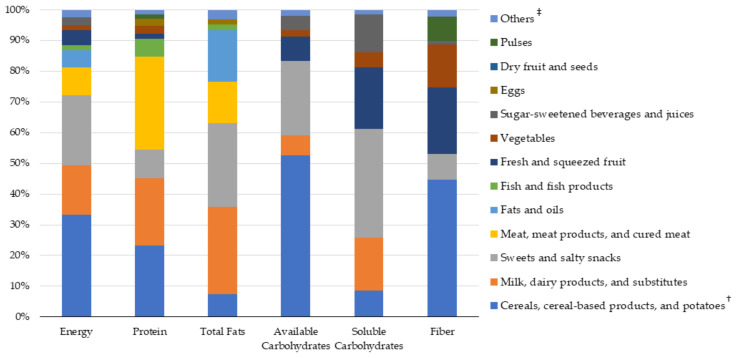
Percentage contribution of the different food groups to total intake of energy and macronutrients. NAC-II, 2014–2016 (*N* = 381). ^‡^ For each variable, the category “Others” included all the remaining food groups. ^†^ “Cereals and cereal-based products” and “Potatoes” were clustered together, as well as “Meat and meat products” and “Cured meat”. The full list of food groups and their contribution to intake of fatty acids, cholesterol, and micronutrients are presented in Supplementary Tables S2 and S3.

Figure 6 shows the percentage contribution of food groups to energy, protein, total fats, available carbohydrates, soluble carbohydrates, and fiber. The full list of food groups and their contribution to intake of fatty acids, cholesterol, and micronutrients are presented in Supplementary Tables S2 and S3.

The main sources of energy intake were “Cereals, cereal-based products and potatoes” (33.2%), “Sweet and salty snacks” (22.6%), and “Milk, dairy products, and substitutes” (16.1%). “Sweet and salty snacks” were the main sources of soluble carbohydrates (35.4%), followed by “Fresh and squeezed fruit” (20.1%), “Milk, dairy products, and substitutes” (17.2%), and “Sugar-sweetened beverages and juices” (12.4%). The major contributors of protein intake were “Meat, meat products, and cured meat” (30.3%), “Cereals, cereal-based products and potatoes” (23.2%), and “Milk, dairy products, and substitutes” (22.0%). “Fish and fish products” contributed to protein intake only for ~6.0%. “Milk, dairy products, and substitutes” (28.5%) and “Sweet and salty snacks” (27.1%) were the main sources of total fats, followed by “Fats and oils” (16.7%) and “Meat, meat products, and cured meat” (13.6%). Finally, the major sources of available carbohydrates and fiber were similar and included “Cereals, cereal-based products and potatoes” (52.6% and 44.7%), “Sweet and salty snacks” (24.2% and 8.4%), “Fresh and squeezed fruit” (8.0% and 21.6%), and “Vegetables” (2.0% and 13.9%). “Pulses” contributed to fiber intake only for the 7.8%.

## 4. Discussion

The current study evaluated nutritional adequacy in 381 7-year-old children from Friuli Venezia Giulia, Italy, who were enrolled within a cohort study aimed at evaluating the effects of mercury on infant neurodevelopment [42]. Results revealed an inadequate intake of key nutrients, as highlighted by standard analyses and the NAR indexes, and suboptimal adequacy of the overall dietary profile, as expressed by the MAR index. In the standard comparison with DRVs, distribution of macronutrient intakes in percentage of energy was unbalanced in favor of protein and fats, with protein intake exceeding the recommendation from 2 to 4 times. Similarly, inadequacy by excess intake was found for most (range: 65.0–84.2%) of the children for soluble carbohydrates, SFAs, and sodium. Within a range of median values between 0.11 and 0.90, the NAR-based analysis further confirmed and allowed to quantify inadequacy by defect for some micronutrients, including vitamin D and folate; it also downgraded evidence on zinc inadequacy, previously emerged in standard DRV-based analysis. A median MAR value of 0.75, with no child reaching the optimal adequacy value of 1, suggested a suboptimal adequacy of the overall diet in the study population.

Considering available carbohydrates, total fats, and protein, most of the children from our sample met the Italian DRVs and showed a NAR index equal to 1. Although apparently reassuring, this hides a substantial unbalance of the overall diet towards total fats and protein. Indeed, in our sample, no child was below the RI lower limit for total fats, and 1 out of 4 children (25.2%) exceeded the upper RI limit. In addition, by calculating child-specific PRI cut-offs for protein, ~63% of the children at least doubled their recommended PRI and ~11% at least tripled it. From a different perspective, the median protein intake of our sample is 55.6 g/d, which is comparable to the daily protein requirement of an adult woman of 60 kg of weight (54 g/day) [38]. The described unbalance towards protein and total fats has been already documented in most of the other Italian [34,35] and European [27,29,30,31] studies on primary school children, except for one older Italian study [32] where available carbohydrates of 8-year-old children reached 60% of total energy intake. In addition, our analysis on food groups suggested that at least 60% of protein daily intake was from animal sources, indicating a low plant-based protein intake, as previously observed in children from the same age in Italy, Spain, and Belgium [37,52,53]. Western dietary pattern, which is high in animal sources, has been previously associated with an increased risk of metabolic syndrome [54].

Still in line with the Italian and European data [24,34,35,55], we observed: 1. an excess intake of SFAs, with 82% of children being above the SDT; and 2. intakes of total PUFAs below the RI lower limit in 96% of the children.

We similarly observed an excess contribution to energy intake from soluble carbohydrates, when using the SDT as the reference cut-off [24,34,35,55]. In addition, 15% of the enrolled children derived at least 25% of their total energy intake from soluble carbohydrates, against recommendations of the Italian Society of Human Nutrition [38], who considered intakes >25% to be at risk for adverse effects on health. Furthermore, no child from our sample had a soluble carbohydrates intake <5 %En, 4 children were < 10 %En vs. 60 (15.8%) < 15 %En, which corresponds to the Italian SDT. This is far from the World Health Organization recommendation to reduce the intake of free sugars to <5 %En, due to their effects on body fat deposition, overweight and obesity, cardiovascular risk, and dental caries [56].

Even if underestimation of sodium is likely to occur in dietary records, our median sodium intake (1.7 g/day) was in line with the one reported by Rosi et al. [35] (1.8 g/day), whereas Verduci et al. [34] reported a lower median intake (1.2 g/day); in the UK-based Avon Longitudinal Study of Parents and Children (ALSPAC) study sex-specific medians were higher (2.1 and 2.3 g/day) and sodium was estimated without considering added salt [30]. In addtion, 65% of our enrolled children had a sodium intake above the SDT [38], without any other possible comparisons except for Verduci et al. [34], where 9% of children were above the SDT, in line with their lower median intake. Major food sources included “Cereal and cereal-based products” (34.4%), followed by “Herbs, spices and added salt” (14.1%), probably due to an increased frequency of consumption of bread substitutes [57] and ready-to-eat products [58,59], which are rich in salt [48]. The “Sweets and salty snacks” food group provided a nonnegligible contribution (8.9%) to sodium intake, with sweets accounting for 92.9% of the food group contribution. This suggests sodium is present in sweets too. Although in “Milk, dairy products, and substitutes” and “Cured meat” salt is traditionally used as a preservative [60], in sweets it is commonly used as a flavor enhancer [60].

Except for iron, copper, and phosphorus, intake of other minerals was generally inadequate (i.e., median intake lower than the corresponding AR) in our sample. Similar conclusions were reached for iron, phosphorus, sodium, calcium, potassium, and zinc, in one or more of the available Italian studies [34,35]. However, generally, higher mean/median intakes were shown in the comparison with previous European studies [30,31,61], as well as with the very detailed but older Italian INRAN-SCAI study [24]. Downgrading evidence on zinc inadequacy from standard DRV-based analysis, the NAR-based approach revealed a modest deviation of zinc intake from the DRVs in most children (median NAR = 0.90). This indicates that dietary inadequacy was not severe in our sample, as also hypothesized for zinc deficiency in serum of European children [62].

Seven out of 12 available vitamins showed a median adequate intake, with five of them (vitamin B2, vitamin B6, vitamin B12, vitamin A, and vitamin C) even reaching the PRI. However, in our analysis we detected a major contribution of food groups of animal origin to vitamin B2 (>60%), vitamin B6 (>43%), and vitamin B12 (100%), as well as to energy (>30%). Among others, the worst degree of inadequacy was observed for folate and vitamin D. Folate median intake was below the AR and 80% of children did not reach it. In Italy and Europe, mean/median intake of folate in primary school children was similarly low [30,31,35,61]. In our sample, the main source of the folate was “cereals and cereal-based products”, where folate intakes may be underestimated due to cooking losses. An inadequate intake of vitamin D was also observed in our sample: no child met the AR, and the median intake of children was 11%, as compared to the AR cut-off value (median NAR = 0.11). This is alarming, but in line with dietary data of other Italian [24,34,35] and European studies [30,31]. Evidence of serum deficiency of vitamin D was also reported in a pediatric population in Italy [63,64], suggesting an increased sun exposure and dietary intake has to be reached.

We did not observe substantial variation in nutrient intake between males and females. Most of the significant differences were found for macronutrients, with the higher available carbohydrates intake in males likely reflected in their higher energy intake, as also found in Verduci et al. [32]. This is in line with previous results from the ALSPAC [30] and from our NAC-II cohort in children at 18 months [45], where, however, soluble carbohydrates were also significantly different between males and females. Only four micronutrients showed a significantly different intake in males and females, but those differences were small and likely to be not nutritionally meaningful.

The major strength of the present work stands in its comprehensive description of dietary intake following different approaches. To our knowledge, we were the first group in Italy to propose the use of NAR and MAR indexes for a quantitative evaluation of dietary adequacy at the nutrient- and overall-diet-levels. We extended the MAR index in two directions: 1. including additional macro- and micronutrients; 2. considering nutrient inadequacy by excess intake together with deficiency in the calculation of the corresponding NARs. We also carried out an influence analysis to assess the importance of single nutrients in the calculation of the MAR index, with reassuring results. We finally summarized information on percentage contribution of selected food groups to macro- and micronutrients, to provide an updated benchmark information for future Italian studies on primary school children. This comprehensive approach has been possible because we collected information based a 3-dDR, which provided a precise quantification of daily food intake [26]. In addition, we referred to the “Food Composition Database for Epidemiological Studies in Italy” [48] to derive intakes of a complete list of 86 macro- and micronutrients. Among the 37 nutrients compared with DRVs, 24 did not show missing values in the BDA. We were only unable to compare four nutrients with the available Italian DRVs [38]: chrome, fluorine, and molybdenum were not provided by the BDA and vitamin K was fraught with so many missing values (86% of the total BDA items) to likely not providing a reliable estimate of its intake.

The current study also has limitations. The cohort was enrolled in the Friuli Venezia Giulia region with a different aim, so generalizability of results to the Italian population of 7-year-old children of the same or next time span is questionable. However, percentages of males and females, prevalence of overweight, as well as percentages of mothers and fathers with a high school diploma were similar to those reported in the national survey “OKkio alla SALUTE” on 8–9-year-old children in 2016, for the Friuli Venezia Giulia region [65] and at the national level [66]. Prevalence of obesity in our sample was in line with data from Friuli Venezia Giulia (5.0%, [65]), but lower than the national-level data (9.3%, [66]). This likely reflects the high frequency of practicing sports and the modest screen time detected in our sample; however, a proper comparison with “OKkio alla SALUTE” was not possible due to differences in questionnaires. Although a dietary record is the gold standard dietary assessment method [26], its use may still lead to biases. Dietary records were filled in by caregivers, who may not be fully aware of child food consumption, especially when the child has lunch at school and/or more than one caregiver is in charge of him/her. Dietary records may be incomplete or inaccurately completed. In case of missing information, standard recipes and standard portion sizes were used. In our 3-dDRs, added salt was not accurately reported by all subjects, and no information on its iodization has been provided. Similarly, water consumption was sporadically reported, leading to possible underestimation of minerals, especially of calcium. An additional source of underestimation of nutrients—common to other studies—included missing data in the food composition tables. Moreover, the use of nutritional labels for the conversion of complex commercial products (~4% of the total food items) may have led to inaccurate estimates of a few macronutrients and/or underestimation of micronutrient intakes. We were also unable to properly estimate child-specific energy requirement due to lack of a dedicated tool to assess physical activity level. In our application, we have to acknowledge that the lack of standardized cut-offs for NAR and MAR have limited our ability to distinguish between modest and severe nutritional inadequacy. Finally, we cannot exclude that inadequacy observed in the present study simply reflected a limited dietary variety [67] or a low adherence to the Mediterranean diet [68,69]. Dietary pattern analysis may provide additional insight into children’s overall dietary behavior and its potential relation with nutritional adequacy.

## 5. Conclusions

In line with previous Italian and European studies on primary school children, the nutritional assessment of a sample of 7-year-old children enrolled in the NAC-II cohort from Friuli Venezia Giulia, Italy, has revealed an unbalanced macronutrient profile towards protein and fats and a suboptimal intake of several macro- and micronutrients. For the first time in an Italian study, our paper has explored the use of two indexes integrating the standard evaluation of nutritional adherence to DRVs. These indexes may provide the basis for targeted public health interventions. They indeed may allow to identify critical nutrients whose intakes have to be modified or subsets of subjects to be targeted within the general population.

## Figures and Tables

**Table 1:** Children’s lifestyle and anthropometric characteristics at 7 years of age. Northern Adriatic Cohort II (NAC-II), 2014–2016 (*N* = 381).

	Median	25th–75th Centile
Child weight at 7 years (kg) ^1^	25.5	22.8–29.2
Child height at 7 years (cm) ^1^	124.3	121.0–128.5
	*N*	%
Sex		
Male	197	51.7
Female	184	48.3
**Weight status ^1^**		
Underweight	9	2.6
Normal weight	255	72.9
Overweight	67	19.1
Obese	19	5.4
**Extra-curricular sport or play activities**		
Never	15	3.9
1–3 days/week	303	79.5
>4 days/week	58	15.2
Not reported	5	1.3
**Videogames activity**		
Never	106	27.8
<1 h/day	172	45.1
1–2 h/day	64	17.6
3 h/day	5	1.3
Not reported	34	8.9
Television use		
Never	15	3.9
<1 h/day	105	27.6
1–2 h/day	233	61.2
3–4 h/day	22	5.8
Not reported	6	1.6
**Food consumption while screen-time activities**		
Yes	103	27.0
No	274	71.9
Not reported	4	1.0
**Caregiver (weekdays) ^2^**		
Mother	326	86.2
Father	146	38.7
Grandparents	135	35.8
School	164	43.4
Baby-sitter	13	3.4
Others	19	5.1

^1^ Anthropometric information was available for 350 children only. ^2^ More than a category was available for responders; therefore, the total does not sum up to 381. In detail, 376 parents selected the “Others” option, 377 parents selected the “Father”, “Grandparents”, and “Baby-sitter” option, 378 parents selected the “Mother” or “School” option.

**Table 2:** Distribution of energy and macronutrient intakes of 7-year-old children in the overall sample and stratified by sex. Northern Adriatic Cohort II (NAC-II), 2014–2016 (*N =* 381).

					Females (*N* = 184)			Males (*N* = 197)			
	Median	25th	75th	DRVs	Median	25th	75th	Median	25th	75th	*p*-Value
Energy (kJ/d)	6291.8	5593.2	6982.2		6139.2	5261	6862.2	6404.4	5773.6	7113.2	0.002 *
Energy (kcal/d)	1503.0	1336.2	1668.0		1466.6	1256.8	1639.3	1530.0	1379.3	1699.3	0.002 *
Protein (g/d)	55.6	47.2	64.3		54.0	46.3	63.4	56.8	48.4	64.5	0.077
Protein (%En)	14.8	13.2	16.5	12–18 %En (RI) ^1^	14.9	13.3	16.9	14.8	13.0	16.3	0.165
Total fats (g/d)	52.2	42.6	61.7		51.1	42.0	60.4	53.3	43.4	63.7	0.084
Total fats (%En)	31.3	27.4	35.1	20–35 %En (RI)	32.0	27.7	35.1	30.8	27.2	35.1	0.309
Saturated fatty acids (g/d)	20.5	16.5	24.5		20.2	14.9	24.0	20.9	17.0	25.0	0.055
Saturated fatty acids (%En)	12.2	10.6	14.0	<10 %En (SDT)	12.2	10.8	14.0	12.2	10.4	14.1	0.865
Monounsaturated fatty acids (g/d)	18.0	14.5	22.1		18.0	14.0	21.7	18.1	14.8	22.1	0.307
Monounsaturated fatty acids (%En)	10.8	9.3	12.6	10–15 %En (RI) ^2^	11.1	9.4	12.7	10.7	9.3	12.4	0.137
Oleic acid (g/d)	16.5	13.4	20.1		16.6	13.2	20.0	16.5	13.8	20.1	0.405
Polyunsaturated fatty acids (g/d)	5.2	4.1	6.6		5.3	4.1	6.7	5.1	4.1	6.5	0.792
Polyunsaturated fatty acids (%En)	3.1	2.5	3.9	5–10 %En (RI)	3.3	2.6	4.0	3.0	2.4	3.7	0.021 *
Arachidonic acid (mg/d)	146.3	95.6	219.7		143.2	96.4	206.5	154.2	94.5	226.9	0.503
Linoleic acid (g/d)	3.9	3.1	5.2		3.9	3.1	5.3	3.8	3.0	5.2	0.509
PUFAs ω-6 (%En)	2.4	2.0	3.2	4–8 %En (RI)	2.6	2.1	3.3	2.3	1.9	3.0	0.007 *
Alpha-linolenic acid (g/d)	0.6	0.4	0.7		0.5	0.4	0.7	0.6	0.5	0.7	0.101
EPA + DHA (mg/d)	61.0	23.7	208.3	250 mg/d (AI)	53.9	24.0	207.3	67.3	23.7	210.0	0.620
PUFAs ω-3 (%En)	0.4	0.3	0.5	0.5–2.0 %En (RI)	0.4	0.3	0.5	0.4	0.3	0.5	0.572
Cholesterol (mg/d)	185.3	143.0	224.8		186	141.9	225.5	183.0	143.3	224.4	0.852
Available carbohydrates (g/d)	197.6	163.7	223.9		189.3	153.0	215.8	204.1	172.0	230.4	<0.001 *
Available carbohydrates (%En)	51.8	48.3	56.6	45–60 %En (RI)	51.3	47.7	55.6	52.5	48.7	57.3	0.095
Soluble carbohydrates (g/d)	72.5	59.0	87.5		71.2	58.6	85.8	74.7	60.1	91.3	0.057
Soluble carbohydrates (%En)	19.4	16.4	23.0	<15 %En (SDT)	19.4	16.3	23.3	19.4	16.4	22.7	0.948
Fiber (g/1000 kcal/d)	7.0	5.7	8.7	8.4 g/1000 kcal (AI)	7.2	5.8	8.9	7.0	5.4	8.5	0.139

^1^ Reference Intake calculated by difference: RI(protein) = 100%- RI(total fats)- RI(available carbohydrates); ^2^ Reference Intake calculated by difference: RI(MUFAs) = RI(total fats)- RI(PUFAs)- SDT(SFAs). Abbreviations: d, day; DRVs, Dietary Reference Values; RI, Reference Intake; SDT, Suggested Dietary Target; AI, Adequate Intake; %En, percentage of daily energy intake; SFAs, saturated fatty acids; MUFAs, monounsaturated fatty acids; PUFAs, polyunsaturated fatty acids; EPA, eicosapentaenoic acid; DHA, docosahexaenoic acid. The two-sample Wilcoxon rank-sum (Mann–Whitney) test was applied to detect any statistical differences between females and males; * *p* < 0.05.

**Table 3:** Distribution of micronutrient intakes of 7-year-old children in the overall sample and stratified by sex. Northern Adriatic Cohort II (NAC-II), 2014–2016 (*N* = 381).

					Females (*N* = 184) (*N* = 197)			Males (*N* = 197)			
	Median	25th	75th	DRVs	Median	25th	75th	Median	25th	75th	*p*-Value
Sodium (g/d)	1.7	1.3	2.1	1.1 g/d (AI); 1.5 g/d (SDT)	1.7	1.3	2.1	1.8	1.4	2.1	0.207
Potassium (g/d)	1.8	1.4	2.1	3 g/d (AI)	1.7	1.4	2.1	1.8	1.5	2.1	0.719
Calcium (mg/d)	537.8	409.6	706.3	900 mg/d (AR); 1100 mg/d (PRI)	515.9	392.4	657.0	567.7	420.1	721.7	0.034 *
Magnesium (mg/d)	83.5	65.5	105.8	130 mg/d (AR); 150 mg/d (PRI)	81.8	61.7	104.2	85.3	67.0	106.0	0.193
Phosphorus (mg/d)	819.0	693.8	966.9	730 mg/d (AR); 875 mg/d (PRI)	778.7	668.5	941.4	846.2	715.7	975.8	0.020 *
Iron (mg/d)	5.9	4.8	7.2	5 mg/d (AR); 13 mg/d (PRI)	5.6	4.6	7.1	6.1	5.0	7.5	0.066
Zinc (mg/d)	6.3	5.3	7.4	7 mg/d (AR); 8 mg/d (PRI)	6.0	5.1	7.3	6.6	5.6	7.4	0.004 *
Selenium (μg/d)	18.5	13.0	28.2	30 μg/d (AR); 34 μg/d (PRI)	17.4	13.2	27.4	19.1	12.9	28.7	0.336
Copper (mg/d)	0.4	0.2	0.5	0.4 mg/d (AR); 0.6 mg/d (PRI)	0.4	0.2	0.5	0.4	0.3	0.5	0.288
Chloride (g/d)	1.3	1.0	1.7	1.7 g/d (AI); 2.3 g/d (SDT)	1.2	1.0	1.7	1.3	1.0	1.7	0.331
Manganese (mg/d)	0.4	0.2	0.6	1.2 mg/d (AI)	0.4	0.2	0.6	0.4	0.2	0.6	0.650
Iodine (μg/d)	75.0	51.8	104.6	100 μg/d (AI)	69.3	47.2	104.8	80.0	55.1	104.6	0.077
Vitamin B1 (mg/d)	0.7	0.6	0.9	0.6 mg/d (AR); 0.8 mg/d (PRI)	0.7	0.6	0.8	0.7	0.6	0.9	0.127
Vitamin B2 (mg/d)	1.1	0.8	1.3	0.7 mg/d (AR); 0.8 mg/d (PRI)	1.0	0.8	1.3	1.1	0.9	1.3	0.014 *
Niacin (mg/d)	9.6	7.7	12.2	9 mg/d (AR); 12 mg/d (PRI)	9.6	7.6	12.1	9.5	7.7	12.3	0.890
Pantothenic acid (mg/d)	2.1	1.6	2.6	3.5 mg/d (AI)	2.2	1.7	2.7	2.1	1.6	2.6	0.979
Vitamin B6 (mg/d)	1.3	1.0	1.5	0.7 mg/d (AR); 0.9 mg/d (PRI)	1.2	1.0	1.5	1.3	1.1	1.5	0.394
Biotin (μg/d)	11.5	8.6	14.7	20 μg/d (AI)	11.5	8.4	14.8	11.5	8.7	14.6	0.929
Folate (μg/d)	160.6	127.5	199.7	210 μg/d (AR); 250 μg/d (PRI)	155.1	125.5	194.1	165.7	135.0	204.2	0.072
Vitamin B12 (μg/d)	2.4	1.8	3.3	1.3 μg/d (AR); 1.6 μg/d (PRI)	2.4	1.7	3.1	2.5	1.9	3.4	0.133
Vitamin A (μg/d) ^1^	603.7	438.8	853.4	350 μg/d (AR); 500 μg/d (PRI)	589.6	421.8	856.2	617.4	450.1	853.1	0.419
Vitamin C (mg/d)	63.2	40.7	98.4	45 mg/d (AR); 60 mg/d (PRI)	61.6	41.0	97.5	63.7	40.4	98.4	0.984
Vitamin D (μg/d)	1.1	0.7	1.5	10 μg/d (AR); 15 μg/d (PRI)	1.0	0.7	1.3	1.1	0.8	1.6	0.142
Vitamin E (mg/d) ^2^	5.1	4.0	6.6	8 mg/d (AI)	5.2	4.1	6.6	5.1	4.0	6.6	0.496

^1^ Expressed as retinol equivalents; ^2^ Expressed as alpha-tocopherol equivalents. The two-sample Wilcoxon rank-sum (Mann–Whitney) test was applied to detect any statistical differences between females and males; * *p* < 0.05. Abbreviation: d, day.

## Data Availability

The data described in the manuscript, in the code book, and in the analytical code will not be made available because we do not have an accessible repository in which to deposit them.

## Supplementary Materials

The following supporting information can be downloaded at: https://www.mdpi.com/article/10.3390/nu14030515/s1, Table S1: Parents general information at delivery. Northern Adriatic Cohort II (NAC-II), 2014–2016 (*N* = 381); Table S2: Percentage contribution of food groups to total intake of fatty acids and cholesterol. NAC-II, 2014–2016 (*N* = 381); Table S3: Percentage contribution of food groups to total intake of micronutrients. NAC-II, 2014–2016 (*N* = 381).
