# Supplementary material for: Adherence to Dietary Recommendations of 7-Year-Old Children from a Birth Cohort in Friuli Venezia Giulia, Italy
Source: Nutrients. 2022 Jan 25;14(3):515. doi: 10.3390/nu14030515 (PMC8838727; doi:10.3390/nu14030515)
Supplement: Supplementary file 1 [file nutrients-14-00515-s001.zip › nutrients-1547104-SI.pdf]

Supplementary Materials

Adherence to Dietary Recommendations of 7-Year-old Children from a Birth Cohort in Friuli Venezia Giulia, Italy

Elisa Giordani, Michela Marinoni, Federica Fiori, Federica Concina, Luca Ronfani, Patrizia Dalmin, Fabio Barbone, Valeria Edefonti and Maria Parpinel

Table S1. Parents general information at delivery. Northern Adriatic Cohort II (NAC-II), 2014–2016 (*N* = 381).

	N	%
**Maternal nationality**		
Italian	353	92.6
Foreign	24	6.3
Not reported	4	1.1
**Maternal marital status**		
Married/living with partner	342	89.7
Separated/divorced	12	3.1
Single/not living with partner	25	6.6
Not reported	2	0.5
**Maternal education**		
Completed primary school	4	1.0
Completed secondary school	57	15.0
Completed high school or equivalent	172	45.1
Bachelor degree or higher	147	38.6
Not reported	1	0.3
**Paternal education**		
Completed primary school	4	1.0
Completed secondary school	106	27.8
Completed high school or equivalent	179	47.0
Bachelor degree or higher	84	22.0
Not reported	8	2.1

Table S2. Percentage contribution of food groups to total intake of fatty acids and cholesterol. NAC-II, 2014–2016 (N = 381).

	%									
	**SFAs**	**MUFAs**	**PUFAs**	**OA**	**LA**	**ALA**	**ARA**	**EPA**	**DHA**	**Chol**
Cereals and cereal-based products	5.4	7.5	**12.9**	7.7	**15.1**	9.3	0.5	5.3	0.0	6.0
Potatoes	0.2	0.0	2.7	0.0	2.7	6.0	0.0	0.0	0.0	0.0
Pulses	0.1	0.1	0.5	0.1	0.4	1.8	0.0	0.0	0.0	0.0
Vegetables	0.2	0.1	1.6	0.1	1.2	6.4	0.0	0.0	0.0	0.0
Fresh and squeezed fruit	0.2	0.2	1.4	0.2	1.3	4.3	0.0	0.0	0.0	0.0
Dry fruit and seeds	0.2	0.8	3.6	0.8	4.0	3.8	0.0	0.0	0.0	0.0
Milk, dairy products and substitutes	**41.2**	**22.6**	8.5	**21.1**	6.5	**32.8**	0.0	0.0	0.0	**24.3**
Meat and meat products	6.0	7.4	**13.1**	7.0	**11.7**	6.1	**48.6**	**20.5**	**12.8**	**21.5**
Cured meat	5.4	8.0	9.7	7.7	**10.1**	5.9	**29.5**	7.5	1.0	6.7
Fish and fish products	0.9	1.3	5.1	1.0	2.1	1.1	9.7	**66.8**	**86.1**	5.9
Eggs	1.6	1.5	2.5	1.5	2.7	0.7	9.9	0.0	0.0	**21.3**
Fats and oils	6.6	**33.0**	**18.8**	**35.2**	**22.8**	9.4	0.1	0.0	0.0	0.0
Sweets and salty snacks	**31.3**	**17.0**	**17.2**	**16.9**	**16.6**	**11.2**	1.6	0.0	0.0	**14.3**
Sugar-sweetened beverages and juices	0.0	0.0	0.1	0.0	0.1	0.1	0.0	0.0	0.0	0.0
Sauces and soups	0.4	0.4	1.8	0.4	2.3	0.1	0.0	0.0	0.0	0.1
Vegetable and meat broths	0.1	0.2	0.2	0.2	0.3	0.2	0.0	0.0	0.0	0.0
Herbs, spices and added salt	0.0	0.0	0.1	0.0	0.1	0.8	0.0	0.0	0.0	0.0
Non-sweetened beverages	0.0	0.0	0.0	0.0	0.0	0.0	0.0	0.0	0.0	0.0

The major food group contributors for each micronutrient were indicated in bold typeface. Abbreviations: SFAs, saturated fatty acids; MUFAs, monounsaturated fatty acids; PUFAs, polyunsaturated fatty acids; OA, oleic acid; LA, linoleic acid; ALA, alpha-linolenic acid; ARA, arachidonic acid; EPA, eicosapentaenoic acid; DHA, docosahexaenoic acid; Chol, cholesterol.

Table S3. Percentage contribution of food groups to total intake of micronutrients. NAC-II, 2014–2016 (*N* = 381).

	**%**																
	**Na**	**K**	**Ca**	**Fe**	**Zn**	**Mg**	**P**	**Se**	**VitB1**	**VitB2**	**VitB3**	**VitB6**	**VitB9**	**VitB12**	**VitC**	**VitD**	**VitE**
Cereals and cereal-based products	**34.4**	**14.5**	5.9	**22.4**	**25.7**	2.6	**17.9**	0.8	**28.7**	**16.9**	**20.1**	**19.4**	**30.6**	0.0	2.5	3.9	9.8
Potatoes	0.2	**8.0**	0.5	2.5	1.1	0.0	1.7	0.0	3.5	1.0	6.3	8.8	5.3	0.0	5.1	0.0	0.3
Pulses	0.2	2.8	1.1	4.3	1.5	0.0	1.9	0.0	5.0	1.6	1.4	1.3	5.2	0.0	2.5	0.0	0.7
Vegetables	2.1	**12.3**	5.0	**12.0**	5.0	4.4	4.1	0.9	6.7	7.7	7.4	**10.2**	**21.1**	0.0	**29.0**	0.6	**18.5**
Fresh and squeezed fruit	0.2	**15.7**	3.5	9.0	2.7	**18.5**	3.0	2.4	7.2	4.7	5.0	**11.3**	7.6	0.0	**34.7**	0.0	7.5
Dry fruit and seeds	0.0	0.4	0.1	0.5	0.6	1.7	0.4	0.2	0.6	0.2	0.3	0.4	0.4	0.0	0.0	0.0	1.2
Milk, dairy products and substitutes	**13.2**	**17.9**	**69.1**	4.7	**22.3**	**34.9**	**36.1**	**21.7**	**11.0**	**39.5**	2.5	**11.4**	**10.8**	**33.9**	2.5	**10.7**	5.8
Meat and meat products	6.6	**11.1**	1.1	**13.6**	**20.7**	**16.6**	**13.4**	**29.4**	**13.9**	**11.2**	**36.6**	**20.0**	3.8	**23.8**	0.0	**22.1**	2.3
Cured meat	**10.8**	2.9	0.4	2.8	7.2	4.0	5.3	4.2	**10.7**	3.0	6.1	7.0	1.4	6.1	0.0	**10.1**	0.2
Fish and fish products	2.5	3.0	1.3	4.5	4.3	5.1	4.3	**35.9**	1.7	2.0	7.5	3.7	1.0	**27.6**	0.2	**22.3**	4.0
Eggs	0.8	0.8	0.9	2.6	2.0	1.6	2.7	2.7	1.3	2.9	0.1	1.0	3.1	8.6	0.0	**14.6**	2.2
Fats and oils	0.1	0.2	0.1	0.5	0.1	0.0	0.0	0.0	0.1	0.1	0.1	0.0	0.0	0.0	0.1	0.0	**32.9**
Sweets and salty snacks	8.9	6.5	8.2	**15.3**	6.0	3.3	8.0	0.1	6.3	7.1	4.2	3.8	4.5	0.1	1.3	**15.5**	9.9
Sugar-sweetened beverages and juices	0.4	2.7	1.4	2.9	0.7	4.5	0.8	0.5	1.1	0.9	1.1	1.3	1.8	0.0	**21.2**	0.0	3.4
Sauces and soups	0.9	0.0	0.0	0.1	0.1	0.0	0.0	0.0	0.0	0.0	0.0	0.0	0.0	0.0	0.0	0.1	1.0
Vegetable and meat broths	4.5	0.3	0.6	0.5	0.1	0.9	0.2	1.1	1.9	0.5	0.8	0.2	2.5	0.0	0.0	0.0	0.0
Herbs, spices and added salt	**14.1**	0.4	0.6	1.6	0.2	0.7	0.1	0.0	0.1	0.2	0.2	0.2	0.4	0.0	0.9	0.0	0.2
Non-sweetened beverages	0.0	0.4	0.0	0.2	0.0	1.1	0.1	0.0	0.1	0.5	0.2	0.0	0.5	0.0	0.0	0.0	0.0

The major food group contributors for each micronutrient were indicated in bold typeface. Abbreviation: Vit, vitamin.
